# The interaction between smoking and HLA genes in multiple sclerosis: replication and refinement

**DOI:** 10.1007/s10654-017-0250-2

**Published:** 2017-06-08

**Authors:** Anna Karin Hedström, Michail Katsoulis, Ola Hössjer, Izaura L. Bomfim, Annette Oturai, Helle Bach Sondergaard, Finn Sellebjerg, Henrik Ullum, Lise Wegner Thørner, Marte Wendel Gustavsen, Hanne F. Harbo, Dragana Obradovic, Milena A. Gianfrancesco, Lisa F. Barcellos, Catherine A. Schaefer, Jan Hillert, Ingrid Kockum, Tomas Olsson, Lars Alfredsson

**Affiliations:** 1 Institute of Environmental Medicine, Karolinska Institutet, Stockholm, Sweden; 2 UCL/Farr Institute of Health Informatics Research, London, UK; 3 Mathematical Statistics, Stockholm University, Stockholm, Sweden; 4 Neuroimmunology Unit, Department of Clinical Neuroscience, Karolinska Institutet, Stockholm, Sweden; 5 Danish Multiple Sclerosis Center, Department of Neurology, Rigshospitalet, University of Copenhagen, Copenhagen, Denmark; 6 Department of Clinical Immunology, Copenhagen University Hospital, Copenhagen, Denmark; 7 Department of Neurology, Oslo University Hospital, Ullevål, Oslo, Norway; 8 Institute of Clinical Medicine, University of Oslo, Oslo, Norway; 9 Department of Neurology, Military Medical Academy, Belgrade, Serbia; 10 Genetic Epidemiology and Genomics Lab, Division of Epidemiology, School of Public Health, University of California, Berkeley, Berkeley, CA 94720-3220 USA; 11 Kaiser Permanente Division of Research, Oakland, CA 94612 USA; 12 Neuroimmunology Unit, Department of Clinical Neuroscience and Center for Molecular Medicine, Karolinska Institutet at Karolinska University Hospital, Solna, Sweden

**Keywords:** Multiple sclerosis, Smoking, HLA, Gene–environment interaction

## Abstract

Interactions between environment and genetics may contribute to multiple sclerosis (MS) development. We investigated whether the previously observed interaction between smoking and HLA genotype in the Swedish population could be replicated, refined and extended to include other populations. We used six independent case–control studies from five different countries (Sweden, Denmark, Norway, Serbia, United States). A pooled analysis was performed for replication of previous observations (7190 cases, 8876 controls). Refined detailed analyses were carried out by combining the genetically similar populations from the Nordic studies (6265 cases, 8401 controls). In both the pooled analyses and in the combined Nordic material, interactions were observed between HLA-DRB*15 and absence of HLA-A*02 and between smoking and each of the genetic risk factors. Two way interactions were observed between each combination of the three variables, invariant over categories of the third. Further, there was also a three way interaction between the risk factors. The difference in MS risk between the extremes was considerable; smokers carrying HLA-DRB1*15 and lacking HLA-A*02 had a 13-fold increased risk compared with never smokers without these genetic risk factors (OR 12.7, 95% CI 10.8–14.9). The risk of MS associated with HLA genotypes is strongly influenced by smoking status and vice versa. Since the function of HLA molecules is to present peptide antigens to T cells, the demonstrated interactions strongly suggest that smoking alters MS risk through actions on adaptive immunity.

**Electronic supplementary material:**

The online version of this article (doi:10.1007/s10654-017-0250-2) contains supplementary material, which is available to authorized users.

## Introduction

Multiple sclerosis (MS) arises from a combination of a complex genetic predisposition and environmental factors. The strongest genetic associations with MS are located within the human leukocyte antigen (HLA) complex [1, 2] whereas genetic regions outside the HLA complex that influence disease susceptibility have a smaller impact on the disease risk [3, 4]. Well-established environmental factors associated with MS risk are Epstein–Barr virus (EBV) infection [5, 6], vitamin D status [7], sun exposure habits [8], adolescent body mass index [9, 10], and smoking [11]. Interactions between environmental factors and genetics are likely to be involved in the etiology of MS. Based on the Swedish project Epidemiological Investigation of Multiple Sclerosis (EIMS), an interaction between smoking and HLA complex genes regarding risk of MS was reported in 2011 [12]. Smoking increased the risk of MS by a factor of 1.5 among those without HLA associated genetic risk and a combination of the genetic risk factors presence of HLA-DRB*15 and absence of HLA-A*02 increased the risk by a factor of 5.0 among non-smokers. However, among smokers with both genetic risk factors, there was a 15-fold increased risk, compared with non-smokers with neither of the genetic risk factors [12]. Using six independent case–control studies from five different countries, we aimed to investigate whether the finding of an interaction between smoking and HLA genotype could be replicated, refined and extended to include other populations.

## Methods

### Study design and data collection

For each study a more detailed description of study design and data collection is presented in supplementary table 1, and a flow chart presenting the falling-off in each study is presented in Table 1.

**Table 1:** Flow chart, Nordic studies

Study	Included in the study	Data on smoking, birth year, and gender. No overlap EIMS/GEMS	Nordic origin	Past smokers excluded	HLA genotype available. Dataset for analysis
EIMS					
Cases	2488	2488	2027	1569	1308
Controls	5433	5433	4243	3379	1858
GEMS					
Cases	6156	6085	5520	4264	3272
Controls	5408	5357	4568	3762	2382
Danish study					
Cases	2048	2048	1832	1520	1474
Controls	4546	4541	4277	3618	3469
Norwegian study					
Cases	332	316	316	278	211
Controls	773	756	748	702	692

### Swedish studies

The first Swedish study was based on EIMS which is an ongoing population-based case–control study, comprising the Swedish general population aged 16–70 years. Incident cases of MS were recruited from neurology clinics, including all university hospitals in Sweden. All cases fulfilled the McDonald criteria [13]. For each case, two controls were randomly selected from the national population register, frequency matched for the case’s age in 5-year age strata, gender and residential area. Ethical approval was obtained from the Regional Ethical Review Board at Karolinska Institutet. Our previous study presenting an interaction between HLA genotype and smoking based on EIMS used study participants recruited between April 2005 and October 2008 [10]. The EIMS replication analysis was restricted to include participants of Nordic origin (Sweden, Norway, or Denmark) recruited between November 2008 and December 2013. When the Nordic studies were combined into one dataset for more detailed analysis, we included EIMS participants of Nordic origin recruited between April 2005 and December 2013. The replication analysis comprised 763 cases and 1037 controls, whereas 1308 cases and 1858 controls were included in the combined Nordic analysis (Table 1).

The second Swedish study was Genes and Environment in Multiple Sclerosis (GEMS) in which prevalent cases, distinct from those in EIMS, were identified from the Swedish National MS-registry [14] and controls were randomly selected from the national population register matched for age, gender, and residential area at the time of disease onset. Ethical approval was obtained from the relevant ethics committee. All cases fulfilled the McDonald criteria [13]. The study participants were recruited between November 2009 and November 2011. The part of GEMS used in this report comprised 3272 prevalent cases and 2382 matched controls (Table 1).

### Danish study

Patients fulfilling the McDonald criteria were recruited from Neurology units in Danish hospitals between October 2009 and December 2014. The control group comprised healthy white Danish blood donors residing in the area of Copenhagen. The controls were matched to the cases by gender and age in 5-year age strata when included in the present study. Informed consent was obtained from all participants and the study was approved by the local Ethics Committee. The part of the Danish study used in this report comprised 1474 prevalent cases and 3469 controls (Table 1).

### Norwegian study

The Norwegian cases were recruited from the Oslo MS Registry [15] and the controls were recruited from the Norwegian Bone Marrow Donor Registry. The cases were diagnosed in accordance with the Poser and/or McDonald criteria [16, 17] and informed written consent was obtained from all participants. The study was approved by the Regional Committee for Medical and Health Research Ethics South East, Norway. The controls were matched to the cases by gender and age in 5-year age strata when included in the present study. The part of the Norwegian study used in this report comprised 211 prevalent cases and 692 controls (Table 1).

### Serbian study

Cases to the Serbian study were recruited at the Military Medical Academy. All patients fulfilled the McDonald criteria. Controls comprised of volunteers from employees of the Military Medical Academy and from the community. The recruitment of cases and controls took place during 2009 and 2010. Ethical approval was obtained by Ethical Committee of Military Medical Academy. In total, 457 cases and 505 controls from the Serbian study was included in the analysis.

### American study

The American case–control study is based on prevalent cases identified among members of Kaiser Permanente Medical Care Plan, Northern California Region (KPNC) using electronic medical records. Controls were randomly selected from KPNC members and were individually matched to cases on gender, birth date, race/ethnicity, and zip code of the case residence. The study protocol was approved by the Institutional Review Boards of the KP Division of Research and the University of California, Berkeley. The part of the KPNC study used in this report comprised 1013 prevalent cases and 794 controls.

### Definition of smoking habits

For each case in all studies but the Serbian and American studies, the time of the initial appearance of MS symptoms was used as an estimate of the disease onset, and the year in which this occurred was defined as the index year. The corresponding controls were given the same index year. Subjects who smoked regularly during the index year were defined as smokers whereas those who had never smoked before or during the index year were defined as never smokers. We have previously demonstrated that the increased risk of developing MS associated with smoking slowly abates after smoking cessation. A decade after stopping smoking, there is no longer an association between smoking and MS risk [11]. Therefore, subjects who had stopped smoking before the index year were excluded. In the Serbian and American study, less detailed data on smoking habits was available and smoking was defined as ever- or never-smoking at the time of inclusion in the study.

### Genotyping and definitions of genetic risk factors

Subjects in all studies were genotyped for HLA-DRB1*15 and HLA-A*02 alleles. Detailed information regarding genotyping in each study is presented in supplementary table 1. The HLA-DRB1*15 allele is associated with an increased risk of developing MS with an OR around 3, and the subjects were classified according to the carriage of any HLA-DRB1*15 allele versus no carriage. The HLA-A*02 allele has protective association to MS. Absence of HLA-A*02 is thus a risk factor of developing the disease, and the participants were classified according to no carriage of any HLA-A*02 allele versus carriage.

### Statistical analysis

Subjects with different genotypes and smoking habits were compared with regard to MS risk, by calculating odds ratios (OR) with 95% confidence intervals (CI) using logistic regression models. When controls are frequency matched to cases, the matching variables should be included in an unconditional logistic model, whereas conditional logistic regression, in which each matched set forms a stratum, should be used when controls are individually matched to cases. However, losses may be substantial when data contain incomplete matched sets. Data from an individual matched case–control study may also be analyzed with unconditional logistic regression as long as the variables used to form the match are included in the model.

In all studies but the Serbian and American studies, we performed both matched and unmatched analyses of the data. However, only the results from the unmatched analyses are presented since these were in close agreement with those from the matched analyses but had higher precision in terms of more narrow confidence intervals. The Serbian data was analyzed using only unconditional logistic regression whereas conditional logistic regression was used when analyzing the American study.

We investigated the gene–gene interaction between HLA-DRB1*15 and absence of HLA-A*02 as well as the interactions between each of these genetic risk factors and smoking. The potential interactions were analysed using departure from additivity of effects [18, 19] and evaluated by calculating AP (attributable proportion due to interaction), RERI (relative excess rate due to interaction), and SI (synergy index) together with 95% confidence intervals, using the delta method [20]. We also studied the total three-way interaction between HLA-DRB1*15, absence of HLA-A*02, and smoking with regard to MS risk, comparing the joint effect of the three risk factors to the situation when each one acts separately, using the total relative excess risk due to interaction (TotRERI_3_), the total attributable proportion (TotAP_3_) and the total synergy index (TotSI_3_);

$$\begin{aligned} {\text{TotRERI}}_{3} \;({\text{X}}_{1} ,\;{\text{X}}_{2} ,\;{\text{X}}_{3} ) & = ({\text{RR}}_{{{\text{X}}_{1} + {\text{X}}_{2} + {\text{X}}_{3} + }} - {\text{RR}}_{{{\text{X}}_{1} - {\text{X}}_{2} - {\text{X}}_{3} - }} ) - ({\text{RR}}_{{{\text{X}}_{1} + {\text{X}}_{2} - {\text{X}}_{3} - }} - {\text{RR}}_{{{\text{X}}_{1} - {\text{X}}_{2} - {\text{X}}_{3} - }} ) \\ & \quad - ({\text{RR}}_{{{\text{X}}_{1} - {\text{X}}_{2} + {\text{X}}_{3} - }} - {\text{RR}}_{{{\text{X}}_{1} - {\text{X}}_{2} - {\text{X}}_{3} - }} ) - ({\text{RR}}_{{{\text{X}}_{1} - {\text{X}}_{2} - {\text{X}}_{3} + }} - {\text{RR}}_{{{\text{X}}_{1} - {\text{X}}_{2} - {\text{X}}_{3} - }} ) \\ & \quad = {\text{RR}}_{{{\text{X}}_{1} + {\text{X}}_{2} + {\text{X}}_{3} + }} - {\text{RR}}_{{{\text{X}}_{1} + {\text{X}}_{2} - {\text{X}}_{3} - }} - {\text{RR}}_{{{\text{X}}_{1} - {\text{X}}_{2} + {\text{X}}_{3} - }} - {\text{RR}}_{{{\text{X}}_{1} - {\text{X}}_{2} - {\text{X}}_{3} + }} + 2 \\ \end{aligned}$$

$${\text{TotAP}}_{3} \;({\text{X}}_{1} ,{\text{X}}_{2} ,{\text{X}}_{3} ) = \frac{{{\text{TotRERI}}_{3} \;({\text{X}}_{1} ,\;{\text{X}}_{2} ,\;{\text{X}}_{3} )}}{{{\text{RR}}_{{{\text{X}}_{1} + {\text{X}}_{2} + {\text{X}}_{3} + }} }}$$

$${\text{TotSI}}_{3} \;({\text{X}}_{1} ,\;{\text{X}}_{2} ,\;{\text{X}}_{3} )\; = \;\frac{{({\text{RR}}_{{{\text{X}}_{1} + {\text{X}}_{2} + {\text{X}}_{3} + }} - 1)}}{{({\text{RR}}_{{{\text{X}}_{1} + {\text{X}}_{2} - {\text{X}}_{3} - }} - 1) + ({\text{RR}}_{{{\text{X}}_{1} - {\text{X}}_{2} + {\text{X}}_{3} - }} - 1) + ({\text{RR}}_{{{\text{X}}_{1} - {\text{X}}_{2} - {\text{X}}_{3} + }} - 1)}}$$

where X_i_+, X_i_− denote the presence (X_i_ = 1) or absence (X_i_ = 0) of X_i_, i = 1, 2, 3, and RR the relative risk for the development of the disease, given the combination of the presence or absence of the risk factors X_1_, X_2_, … X_3_, as compared to their absence [21]. These measures of interaction between the three factors combine all two-way interactions, when the 3rd risk factor is absent, as well as the three-way interaction, hence called total interaction. We also calculated measures of the three-way interaction where the influence from the possible two-way interactions between the three factors was removed (denoted RERI_3_, AP_3_ and SI_3_) [Katsoulis M and Bamia C. Moving from two- to multi-way interactions among binary factors on the additive scale. Under revision in Am J Epidemiol].

When the results from all six studies were pooled together, study specific results (adjusted for age and gender) were pooled together by utilising a model allowing for random effects. When the Nordic studies were combined into one, the analyses were adjusted for age, gender and study.

In EIMS and GEMS, the interaction analyses were further adjusted for a large number of potential confounding variables described in supplementary table 2. Adjustment for these factors had minor influence on the results. The analyses were conducted using Statistical Analysis System (SAS) version 9.2 and Stata Statistical Software, release 11 (StataCorp. 2009, StataCorp LP).

## Results

The increased MS risk associated with smoking was similar in all studies. HLA-DRB1*15 increased the risk of MS in all studies, whereas HLA-A*02 was associated with a decreased MS risk in all studies but the Serbian study (supplementary table 3). In the pooled analyses there was an interaction between HLA-DRB*15 and absence of HLA-A*02 and between smoking and each of the genetic risk factors (Table 2). The previously reported interaction between HLA-DRB1*15 and HLA-A*02 [12] was replicated in all studies, except for the Serbian study. An interaction between smoking and HLA-DRB1*15 was observed in all studies. With regard to the interaction between smoking and absence of HLA-A*02, an interaction was observed only in the Nordic studies. The results from each of the separate studies are presented in supplementary table 4 whereas the main effects of the HLA genotypes and smoking for each cohort are presented in Table 3.

**Table 2:** Replication and extension of previous results

DR15+	A2−	ca/co^a^	OR (95% CI)^b^	*p*	AP
OR with 95% CI of developing MS for subjects categorized by HLA-DRB1*15 and HLA-A*02. Attributable proportion due to interaction between HLA-DRB1*15 and HLA-A*02					
−	−	1232/3354	1.0 (–)	1.0 (–)	
−	+	1734/2881	1.6 (1.4–1.8)	<0.0001	
+	−	1864/1457	3.6 (3.2–4.0)	<0.0001	
+	+	2360/1184	5.7 (5.2–6.3)	<0.0001	0.34 (0.29–0.39), *p* = 0.0004

A2−	Smoking	ca/co^a^	OR (95% CI)^c^	*p*	AP
OR with 95% CI of developing MS for subjects categorized by HLA-A*02 and smoking. Attributable proportion due to interaction between HLA-A*02 and smoking					
−	−	1517/3181	1.0 (−)		
−	+	1579/1630	3.2 (3.6–4.0)	<0.0001	
+	−	2116/2764	1.8 (1.5–2.1)	<0.0001	
+	+	1978/1301	5.4 (4.4–6.6)	<0.0001	0.21 (0.16–0.27), *p* < 0.0001

DR15+	Smoking	ca/co^a^	OR (95% CI)^d^	*p*	AP
OR with 95% CI of developing MS for subjects categorized by HLA-DRB1*15 and smoking. Attributable proportion due to interaction between HLA-DRB1*15 and smoking					
−	−	1435/4164	1.0 (–)		
−	+	1531/2071	3.3 (2.7–3.9)	<0.0001	
+	−	2198/1781	3.6 (3.0–4.3)	<0.0001	
+	+	2026/860	10.4 (8.5–12.8)	<0.0001	0.35 (0.30–0.40), *p* < 0.0001

All studies pooled together

^a^Number of exposed cases and controls

^b^Adjusted for age, gender, and smoking

^c^Adjusted for age, gender, and HLA-DRB1*15

^d^Adjusted for age, gender, and HLA-A*02

**Table 3:** Two way interactions on the additive scale between HLA-DRB1*15 and absence of HLA-A*02, between absence of HLA-A*02 and smoking, and between HLA-DRB1*15 and smoking

DR15	A2−	Smoking	AP^a^	AP^b^	AP^c^	AP^d^	AP^e^	AP^f^
x	x		0.4 (0.2–0.5)	0.2 (0.04–0.3)	0.3 (0.1–0.4)	0.5 (0.2–0.7)	−0.3 (−1 to 0.4)	0.5 (0.3–0.7)
	x	x	0.3 (0.1–0.5)	0.2 (0.008–0.3)	0.2 (0.005–0.3)	0.3 (0.0–0.7)	0.2 (−0.09 to 0.6)	0.0 (−0.3 to 0.3)
x		x	0.3 (0.08–0.4)	0.2 (0.06–0.3)	0.4 (0.3–0.5)	0.3 (0.05–0.6)	0.4 (0.2–0.7)	0.4 (0.1–0.6)

Attributable proportion due to interaction with 95% CI

^a^EIMS, study

^b^GEMS

^c^Danish study

^d^Norwegian study

^e^Serbian study

^f^American study

By combining the Nordic datasets (6265 cases and 8401 controls), we show that interactions take place between both HLA-DRB1*15 and HLA-A*02 regardless of smoking status (Table 4A), between HLA-A*02 and smoking regardless of HLA-DRB1*15 status (Table 4B), and between HLA-DRB1*15 and smoking regardless of HLA-A*02 status (Table 4C). In all Nordic studies, an overall interaction was observed between these three risk factors (Table 5). However, when the influence from two way interactions was removed, the remaining three way interaction was only significant in the EIMS study, and marginally significant in the combined Nordic study (Table 6).

**Table 4:** Combined study using participants of Nordic origin

DR15+	A2−	ca/co^a^	OR (95% CI)^b^	*p*	AP
*A. OR with 95% CI of developing MS for subjects categorized by HLA*-DRB1*15 and HLA-A*02, in total and stratified by smoking status. Attributable proportion due to interaction between HLA-DRB1*15 and HLA-A*02					
Total					
−	−	991/3192	1.0 (–)		
−	+	1428/2639	1.8 (1.6–1.9)	<0.0001	
+	−	1732/1439	3.9 (3.5–4.3)	<0.0001	
+	+	2114/1131	6.1 (5.5–6.8)	<0.0001	0.2 (0.1–0.4), *p* < 0.0001

DR15+	A2−	ca/co^a^	OR (95% CI)^c^	*p*	AP
Never smokers					
−	−	490/2163	1.0 (–)		
−	+	749/1861	1.8 (1.5–2.0)	<0.0001	
+	−	922/975	4.1 (3.6–4.7)	<0.0001	
+	+	1152/787	6.4 (5.6–7.3)	<0.0001	0.2 (0.1–0.3), *p* < 0.0001

DR15+	A2−	ca/co^a^	OR (95% CI)^c^	*p*	AP
Current smokers					
−	−	501/1029	1.0 (–)		
−	+	679/778	1.8 (1.5–2.1)	<0.0001	
+	−	810/464	3.6 (3.1–4.2)	<0.0001	
+	+	962/344	5.8 (4.9–6.8)	<0.0001	0.2 (0.1–0.4), *p* < 0.0001

A2−	Smoking	ca/co^a^	OR (95% CI)^d^	*p*	AP
*B. OR with 95% CI of developing MS for subjects categorized by HLA*-A*02 and smoking, in total and stratified by HLA-DRB1*15 status. Attributable proportion due to interaction between HLA-A*02 and smoking					
Total					
−	−	1412/3138	1.0 (–)		
−	+	1311/1493	2.0 (1.8–2.2)	<0.0001	
+	−	1901/2648	1.6 (1.5–1.8)	<0.0001	
+	+	1641/1122	3.5 (3.1–3.9)	<0.0001	0.2 (0.1–0.3), *p* < 0.0001

A2−	Smoking	ca/co^a^	OR (95% CI)^c^	*p*	AP
HLA-DRB1*15 negative					
−	−	490/2163	1.0 (–)		
−	+	501/1029	2.1 (1.8–2.5)	<0.0001	
+	−	749/1861	1.8 (1.5–2.0)	<0.0001	
+	+	679/778	3.8 (3.3–4.4)	<0.0001	0.2 (0.1–0.3), *p* < 0.0001

A2−	Smoking	ca/co^a^	OR (95% CI)^c^	*p*	AP
HLA-DRB1*15 positive					
−	−	922/975	1.0 (–)		
−	+	810/464	1.9 (1.6–2.2)	<0.0001	
+	−	1152/787	1.6 (1.4–1.8)	<0.0001	
+	+	962/344	3.1 (2.7–3.7)	<0.0001	0.2 (0.08–0.3), *p* = 0.002

DR15+	Smoking	ca/co^a^	OR (95% CI)^e^	*p*	AP
*C. OR with 95% CI of developing MS for subjects categorized by HLA*-DRB1*15 and smoking, in total and stratified by HLA-A*02 status. Attributable proportion due to interaction between HLA-DRB1*15 and smoking					
Total					
−	−	1239/4024	1.0 (–)	–	
+	+	1180/1807	2.1 (2.0–2.4)	<0.0001	
+	−	2074/1762	3.8 (3.5-4.2)	<0.0001	
+	+	1772/808	7.4 (6.7-8.3)	<0.0001	0.3 (0.26–0.4), *p* < 0001

DR15+	Smoking	ca/co^a^	OR (95% CI)^c^	*p*	AP
HLA-A*02 positive					
−	−	490/2163	1.0 (–)		
−	+	501/1029	2.1 (1.9–2.5)	<0.0001	
+	−	922/975	4.1 (3.6–4.7)	<0.0001	
+	+	810/464	7.7 (6.6–8.9)	<0.0001	0.3 (0.2–0.4), *p* < 0.0001

DR15+	Smoking	ca/co^a^	OR (95% CI)^c^	*p*	AP
HLA-A*02 negative					
−	−	749/1861	1.0 (–)		
−	+	679/778	2.2 (1.9–2.5)	<0.0001	
+	−	1152/787	3.6 (3.2–4.1)	<0.0001	
+	+	344/962	7.2 (6.2–8.4)	<0.0001	0.3 (0.2–0.4), *p* < 0.0001

^a^Number of exposed cases and controls

^b^Adjusted for age, gender, smoking, and study

^c^Adjusted for age, gender, and study

^d^Adjusted for age, gender, HLA-DRB1*15, and study

^e^Adjusted for age, gender, HLA-A*02, and study

**Table 5:** Combined two- and three-way interaction on the additive scale between HLA-DRB1*15, absence of HLA-A*02, and smoking

Study	TotAP_3_	TotRERI_3_	TotSI_3_
EIMS	0.6 (0.4–0.7)	6.7 (3.0–10.3)	2.9 (1.9–4.4)
GEMS	0.3 (0.2–0.5)	3.5 (1.3–5.7)	1.6 (1.2–2.1)
Danish study	0.6 (0.5–0.7)	10.8 (6.5–15.0)	2.8 (2.1–3.7)
Norwegian study	0.7 (0.5–0.9)	12.6 (1.1–24.2)	3.5 (1.6–8.0)
Nordic studies combined	0.5 (0.46–0.61)	6.8 (5.0–8.5)	2.4 (2.0–2.8)

Total attributable proportion due to interaction, relative excess risk and synergy index due to interaction, with 95% CI

**Table 6:** Three way interaction on the additive scale between HLA-DRB1*15, absence of HLA-A*02 and smoking

Study	AP3	RERI3	SI3
EIMS	0.4 (0.1–0.6)	4.4 (0.3–8.4)	1.7 (1.1–2.8)
GEMS	−0.02 (−0.3 to 0.3)	−0.2 (−3.1 to 2.6)	1.0 (0.7–1.3)
Danish study	0.09 (0.2–0.3)	1.6 (−3.4 to 6.5)	1.1 (0.8–1.5)
Norwegian study	0.2 (−0.3 to 0.7)	4.1 (−7.9 to 16.1)	1.3 (0.6–2.8)
Nordic studies combined	0.2 (0.002–0.3)	1.9 (−0.16 to 3.92)	1.2 (1.0–1.4)

Total attributable proportion due to interaction, relative excess risk and synergy index due to interaction, with 95% CI

All analyses were adjusted for age and gender. Smoking has previously been found to correlate with exposure to passive smoking, snuff use, and to a minor extent, alcohol consumption. Therefore, we also present the results in EIMS and GEMS with adjustment for these factors. We further adjusted for an additional number of potential confounding factors including adolescent body mass index, sun exposure habits during the last 5 years, a history of mononucleosis, educational level, and socioeconomic status. However, all potential confounding variables only had minor influence on the results and were not retained when the Nordic studies were combined into one.

## Discussion

The previously reported interaction between smoking and HLA genotype was replicated and extended to include other populations. By combining the genetically similar populations from the Nordic studies we had the opportunity to study the interaction between smoking, HLA-DRB1*15 and absence of HLA-A*02 in detail. Two way interactions were observed between each combination of the three variables, invariant over categories of the third. Further, there was also a three way interaction between the risk factors in the EIMS study, and a marginally significant three way interaction for all the Nordic studies combined. The difference in MS risk between the extremes was considerable; smokers carrying HLA-DRB1*15 and lacking HLA-A*02 had a 13-fold increased risk compared with never smokers without these genetic risk factors (OR 12.7, 95% CI 10.8–14.9).

An interaction between smoking and absence of HLA-A*02 was observed only in the Nordic studies. No interaction between smoking and absence of HLA-A*02 was observed in the Serbian or American cohort. This could be due to chance, differences in life style habits or in genetic background. Notably, the HLA-A*02 protective association to MS vary considerably between different populations [3, 22].

Since use of oral tobacco in the form of moist snuff is not associated with increased risk of MS [23], the critical effects of smoking may be caused by irritation in the lungs. A similar interaction has been observed between passive smoking and the same MS risk HLA genes with regard to MS risk [24]. Thus, lung-irritation displays a considerably higher association with MS among people with a genetic susceptibility to the disease. In addition, non-specific lung-irritation due to organic solvents is also associated with an increased risk of MS as demonstrated in a meta-analysis [25].

There are several potential mechanisms that can be involved in a casual role of smoking. Smoke-induced lung-irritation causes increased pro-inflammatory cell activation and induces post-translational modifications of proteins in the lungs [26–28]. Altered self through peptide modifications may lead to a bypass of thymic tolerance and triggering of autoimmune disease, as has been experimentally demonstrated for myelin oligodendrocyte glycoprotein [29]. Interestingly, the lungs contribute to the T cell activation and migration that are required for experimental autoimmune encephalomyelitis (EAE) initiation [30]. Potentially autoaggressive effector and memory cells are also present and available for triggering in the lungs. In EAE studies, these cells strongly proliferate after local stimulation of the lungs and, after assuming migratory properties, enter the central nervous system (CNS) and induce autoimmune responses [30]. Any CNS autoreactive T cells present in the lung could thus be activated by the proinflammatory action of smoking.

The specific interactions may shed light on potential disease mechanisms for MS. Preferences in peptide binding by allelic variants of class II molecules are likely to be critical for the HLA class II influences on autoimmune diseases [31]. The interaction between smoking and HLA genotype with regard to MS risk is consistent with class II allele specific recognition of particular altered self peptides in the lungs, with ensuing organ specific inflammatory disease depending on preferential peptide binding by allelic variants of class II molecules. Similarly, carriage of the shared epitope, comprising HLA-DRB1*04, interacts with smoking to increase the risk of rheumatoid arthritis [32]. We thus hypothesize that smoking may contribute to the activation of autoaggressive T cells in the lungs and subsequently lead to MS in people with a genetic susceptibility to the disease. Interpretation of the protective association of HLA-A*02 in MS is more challenging. A role for CD8+ T cells is supported by histochemical studies of MS brain lesions where CD8+ T cells dominate over CD4+ T cells [33]. Class I molecules present antigen to CD8+ T cells which can be cytotoxic, but also convey suppressor functions through production of molecules such as TGF β [34]. Class I allele specific suppression mediated by CD8+ cells and TGF beta has been demonstrated in EAE [35–37]. However, further research on CD8 + cells in relation to allelic influences is strongly warranted.

Approximately 20% of all MS cases in Sweden has been estimated to be attributable to active or passive smoking, whereas 41% of the cases among those carrying HLA-DRB1*15 but lacking HLA-A*02 were attributable to smoking [38]. From a public health perspective, the impact of smoking and passive smoking on MS risk is thus considerable and preventive measures in order to reduce tobacco smoke exposure are therefore essential.

In all studies, information regarding smoking habits was collected retrospectively. The EIMS study was designed as a case–control study with incident cases in order to minimize recall bias, whereas the other studies used prevalent cases. The questionnaires in all Nordic studies contained a wide range of questions regarding many potential environmental risk factors and no section in the questionnaires was given prime focus. Furthermore, in the Nordic studies as well as in the American study, exposure information from cases and controls was obtained in an identical way from cases and controls.

A potential selection bias may arise when recruiting cases and controls. Considering the structure of the public health care system in Sweden, which provides equal free of charge access to medical services for all citizens, it is likely that almost all cases of MS are referred to neurological units. However, some cases may not have been asked to participate in EIMS due to the attending neurologists’ lack of time. In GEMS and the Norwegian study, cases were identified through MS registers in each country and these registers may not have covered all prevalent cases. In the Danish study, the majority of cases were randomly recruited by neurologists from hospitals with MS Centers in Denmark and the cases may not be representative for the general MS population. However, the recruitment of cases was probably not affected by smoking or HLA genotype.

In the Swedish studies, the problem of selection bias was minimized by the population-based design and even though there was a relatively high proportion of non-responders among the controls, this bias is probably modest because the prevalence of life style factors, such as smoking and alcohol consumption, among the controls was consistent with that of the general population in similar ages [39]. The distribution of socioeconomic status among controls was also in line with that of the general population [40].

Furthermore, there were no significant differences with respect to age, gender, or smoking habits between those who provided a blood sample and those who did not, indicating that selection bias did not take place in this step. We consider it unlikely that our main finding of an interaction between MS risk HLA genotypes and exposure to smoking would be affected by bias to a large extent, especially since such a bias would then depend on HLA types.

In the Danish study, the control group comprised healthy white Danish blood donors and in the Norwegian study, controls were selected from the National Bone Marrow Donor Registry. In these studies, a selection bias towards a more than normally healthy group may therefore have occurred. However, educational level and autoimmune co-morbidity were similar among Norwegian cases and controls, indicating that the groups were comparable with regard to socioeconomic status and thus probably also general [40]. The proportion of regular smokers among the controls was similar to the frequency in the general Norwegian population, arguing for that the controls were representative with regard to lifestyle factors.

In conclusion, an interaction between smoking and HLA-DRB1*15 was observed in all cohorts, whereas an interaction between smoking and absence of HLA-A*02 was observed only in the Nordic cohorts. The combined Nordic studies allowed detailed analyses of the interaction between the three studied factors, and in addition to significant two way interactions between each combination of the three variables, invariant over categories of the third, there was a significant three way interaction between smoking, HLA-DRB1*15, and absence of HLA-A*02. We hypothesize that smoke-induced lung-irritation may trigger autoaggressive T cells in the lungs or post-translationally modify peptides that are cross-reactive with CNS antigens, promoting a CNS-directed autoaggressive immunity that results in MS.

## Electronic supplementary material

Below is the link to the electronic supplementary material.

Supplementary material 1 (DOC 39 kb)

Supplementary material 2 (DOC 26 kb)

Supplementary material 3 (DOC 26 kb)

Supplementary material 4 (DOC 54 kb)

## References

[1] Lincoln MR, Montpetit A, Cader MZ, Saarela J, Dyment DA, Tiislar M (2005). A predominant role for the HLA class II region in the association of the MHC region with multiple sclerosis. Nat Genet.

[2] Brynedal B, Duvefelt K, Jonasdottir G, Roos IM, Akesson E, Palmgren J (2007). HLA-A confers an HLA-DRB1 independent influence on the risk of multiple sclerosis. PLoS ONE.

[3] Sawcer S (2011). Genetic risk and a primary role for cell-mediated immune mechanisms in multiple sclerosis. Nature.

[4] International Multiple Sclerosis Genetics Consortium (2013). Analysis of immune-related loci identifies 48 new susceptibility variants of multiple sclerosis. Nat Genet.

[5] Levin LI (2010). Primary infection with the Epstein–Barr virus and risk of multiple sclerosis. Ann Neurol.

[6] Handel AE, Williamson AJ, Disanto G, Handunnetthi L, Giovannoni G, Ramagopalan SV (2010). An updated meta-analysis of risk of multiple sclerosis following infectious mononucleosis. PLoS One.

[7] Simon KC, Munger KL, Ascherio A (2012). Vitamin D and multiple sclerosis: epidemiology, immunology, and genetics. Curr Opin Neurol.

[8] Bäärnhielm M, Hedström AK, Kockum I, Sundqvist E, Gustafsson SA, Hillert J, Olsson T, Alfredsson L (2012). Sunlight is associated with decreased multiple sclerosis risk: no interaction with human leukocyte antigen-DRB1*15. Eur J Neurol.

[9] Munger KL, Chintnis T, Ascherio A (2009). Body size and risk of MS in two cohorts of US women. Neurology.

[10] Hedström AK, Olsson T, Alfredsson L. High body mass index before age 20 is associated with increased risk for multiple sclerosis in both men and women. Mult Scler. 2012;18:1334–36. 10.1177/1352458512436596 22328681

[11] Hedström AK, Hillert J, Olsson T, Alfredsson L (2013). Smoking and multiple sclerosis susceptibility. Eur J Epidemiol.

[12] Hedström AK, Sundqvist E, Bäärnhielm M (2011). Smoking and two human leukocyte antigen genes interact to increase the risk for multiple sclerosis. Brain.

[13] Thompson AJ, Montalban X, Barkhof F, Brochet B, Filippi M, Miller DH, Polman CH, Stevenson VL, McDonald WI (2000). Diagnostic criteria for primary progressive multiple sclerosis: a position paper. Ann Neurol.

[14] Hillert J, Stawiarz L (2015). The Swedish MS registry—clinical support tool and scientific resource. Acta Neurol Scand Suppl.

[15] Smestad C, Sandvik L, Holmoy T (2008). Marked differences in prevalence of multiple sclerosis between ethnic groups in Oslo, Norway. J Neurol.

[16] Poser CM, Paty DW, Scheinberg L (1983). New diagnostic criteria for multiple sclerosis: guidelines for research protocols. Ann Neurol.

[17] Polman CH, Reingold SC, Banwell B (2011). Diagnostic criteria for multiple sclerosis: 2010 revisions to the McDonald criteria. Ann Neurol.

[18] Rothman KJ, Greenland S, Lash TL (2008). Modern epidemiology.

[19] VanderWeele TJ (2009). Sufficient cause interactions and statistical interactions. Epidemiology.

[20] Rothman KJ, Greenland S, Walker AM (1980). Concepts of interaction. Am J Epidemiol.

[21] Petti S, Masood M, Scully C (2013). The magnitude of tobacco smoking-betel quid chewing-alcohol drinking interaction effect on oral cancer in South-East Asia. A meta-analysis of observational studies. PLoS ONE.

[22] van der Mei I, Lucas RM, Taylor BV (2016). Population attributable fractions and joint effects of key risk factors for multiple sclerosis. Mult Scler.

[23] Hedström AK, Hillert J, Olsson T, Alfredsson L (2013). Nicotine might have a protective effect in the etiology of multiple sclerosis. Mult scler.

[24] Hedström AK, Bomfim IL, Barcellos LF (2014). Interaction between passive smoking and two HLA genes with regard to MS risk. Epidemiology.

[25] Barragán-Martínez C, Speck-Hernández CA, Montoya-Ortiz G, Mantilla RD, Anaya JM, Rojas-Villarraga A (2012). Organic solvents as risk factor for autoimmune diseases: a systematic review and meta-analysis. PLoS ONE.

[26] Doyle HA, Mamula MJ (2002). Posttranslational protein modifications: new flavors in the menu of autoantigens. Curr Opin Rheumatol.

[27] Cloos PA, Christgau S (2004). Post-translational modifications of proteins: implications for aging, antigen recognition, and autoimmunity. Biogerontology.

[28] Makrygiannakis D, Hermansson M, Ulfgren AK, Nicholas AP, Zendman AJ, Eklund A (2008). Smoking increases peptidylarginine deiminase 2 enzyme expression in human lungs and increases citrullination in BAL cells. Ann Rheum Dis.

[29] Lucca LE, Axisa PP, Aloulou M, et al. Myelin oligodendrocyte glycoprotein induces incomplete tolerance of CD4+ T cells specific for both a myelin and a neuronal self antigen in mice. Eur J Immunol. 2016;46:2247–59. 10.1002/eji.201646416 27334749

[30] Odoardi F, Sie C, Streyl K (2012). T cells become licensed in the lung to enter the central nervous system. Nature.

[31] Olsson T, Hillert J (2008). The genetics of multiple sclerosis and its experimental models. Curr Opin Neurol.

[32] Klareskog L, Stolt P, Lundberg K, Källberg H, Bengtsson C, Grunewald J (2006). A new model for an etiology of RA; Smoking may trigger HLA-DR (SE)-restricted immune reactions to autoantigens modified by citrullination. Arthritis Rheum.

[33] Friese MA, Fugger L (2005). Autoreactive CD8+ T cells in multiple sclerosis: a new target for therapy?. Brain.

[34] Friese MA, Flugger A (2008). Opposing effects of HLA class I molecules in tuning autoreactive CD8+ T cells in multiple sclerosis. Nat Med.

[35] Mustafa M, Vingsbo C, Olsson T (1993). The major histocompatibility complex influences myelin basic protein 63-88-induced T cell cytokine profile and experimental autoimmune encephalomyelitis. Eur J Immunol.

[36] Mustafa M, Vingsbo C, Olsson T (1994). Protective influences on experimental autoimmune encephalomyelitis by MHC class I and class II alleles. J Immunol.

[37] Issazadeh S, Kjellen P, Olsson T (1997). Major histocompatibility complex-controlled protective influences on experimental autoimmune encephalomyelitis are peptide specific. Eur J Immunol.

[38] Hedström AK, Olsson T, Alfredsson L (2016). Smoking is a major preventable risk factor for multiple sclerosis. Mult Scler.

[39] Internet-based information. www.scb.se. Accessed 25 Feb 2006.

[40] Gustavsen MW (2014). Environmental exposures and the risk of multiple sclerosis investigated in a Norwegian case–control study. BMC Neurol.

