# Supplementary material for: The interaction between smoking and HLA genes in multiple sclerosis: replication and refinement
Source: Eur J Epidemiol. 2017 Jun 8;32(10):909–19. doi: 10.1007/s10654-017-0250-2 (PMC5680370; doi:10.1007/s10654-017-0250-2)
Supplement: Supplementary file 1 — Supplementary material 1 (DOC 39 kb) [file 10654_2017_250_MOESM1_ESM.doc]

# Supplementary table 1

**Detailed description of study design and data collection**

**Swedish studies**

The ongoing project Epidemiological Investigation of Multiple Sclerosis (EIMS) is a population-based case-control study on environmental and genetic risk factors for MS. The study group comprises the Swedish population aged 16-70 years. In total, there are today 40 neurology clinics that recruit incident cases of MS to the study, including all university hospitals in Sweden. All cases had been diagnosed by their treating neurologist according to the McDonald criteria^1^. For each case, two controls were randomly selected from the national population register, frequency matched for the case's age in 5-year age strata, gender and residential area.

In EIMS, information regarding environmental exposures and life-style factors such as smoking was collected using a standardised questionnaire. The response rate was 93% for cases and 73% for controls. The EIMS replication analysis was restricted to include participants of Nordic origin (Sweden, Norway, or Denmark) recruited between November 2008 and December 2013. A previously published study presenting an interaction between HLA genotype and smoking based on EIMS used study participants recruited between April 2005 and October 2008^2^. When the Nordic studies were combined into one dataset for more detailed analysis, we included EIMS participants of Nordic origin recruited between April 2005 and October 2008.

The time of the initial appearance of MS symptoms was used as an estimate of the disease onset, and the year in which this occurred was defined as the index year. The corresponding controls were given the same index year. Smoking habits were only considered before the index year. We have previously demonstrated that the increased risk of developing MS associated with smoking slowly abates after smoking cessation. A decade after stopping smoking, there is no longer an association between smoking and MS risk^3^. Therefore, past smokers were excluded. All participants were asked to provide a blood or saliva sample and those who declined to donate blood or saliva were excluded. For EIMS participants included between April 2005 and October 2008, HLA-DRB1 and HLA-A genotypes were obtained as previously described^4^. For EIMS participants recruited between November 2008 and December 2013 genotyping was performed on the MS replication chip^5^ which is based on an Illumina exome chip to which approximately 100,000 custom markers were added and HLA was then imputed with HLA*IMP:02^6^. The replication analysis comprised 763 cases and 1037 controls, whereas 1308 cases and 1858 controls were included in the combined Nordic analysis.

The second Swedish study was GEMS (Genes and Environment in Multiple Sclerosis) in which prevalent cases, distinct from those in EIMS, were identified from the Swedish National MS-registry^7^ and controls were randomly selected from the national population register matched for age, gender, and residential area at the time of disease onset. Ethical approval was obtained from the relevant ethics committee. All cases fulfilled the McDonald criteria^2^.

GEMS used a similar questionnaire as the one used in EIMS, and the questions on smoking habits were identical as those in the EIMS questionnaire. The study participants were recruited between November 2009 and November 2011. The response rate was 82% for the cases and 66% for the controls. The time of the initial appearance of MS symptoms was used as an estimate of the disease onset, and the year in which this occurred was defined as the index year. The corresponding controls were given the same index year. Those who had stopped smoking before the index year were excluded. Subjects of non-Nordic origin and those who had not answered the questions on smoking habits were excluded, as were those who did not provide a blood sample. Genotyping was performed on the MS replication chip^5^ which is based on an Illumina exome chip to which approximately 100,000 custom markers were added and HLA was then imputed with HLA*IMP:02^6^. The part of GEMS used in this report comprised 3272 prevalent cases and 2382 matched controls.

**Danish study**

Patients fulfilling the McDonald criteria were recruited from Neurology units in Danish hospitals. The majority of patients were recruited in the area of Copenhagen. The control group comprised healthy white Danish blood donors residing in the area of Copenhagen. The participants in the Danish study were not controlled for geographic location since Denmark is a small country with 5 million inhabitant located on a compact geographic location on latitude 54^0^-57^0^. The controls were matched to the cases by gender and age in five-year age strata at inclusion in the study. Written informed consent was obtained from all MS patients and approved by the local Ethic Committee (KF-01 314009). With regard to the blood donors, informed consent was obtained through their participation in ”The Danish Blood Donor Study” approved by the local Ethics Committee (M-20090237).

All participants in the Danish study filled out a questionnaire on life style and environmental exposures, adapted from the GEMS study. Of the invited cases, 74% accepted participation in the study between October 2009 and December 2014. Controls were recruited as part of the Danish Blood Donor Study from five major donor locations in the period of October 2012 to December 2014. The participation rate among controls was estimated to be approximately 83%. Subjects of non-Nordic origin were excluded. The time of the initial appearance of MS symptoms was used as an estimate of the disease onset, and the year in which this occurred was defined as the index year. The corresponding controls were given the same index year. Those who had smoked before the index year were excluded. HLA was imputed in the Danish study using the software HLA*IMP:02^6^ based on genotypes obtained as described^8^. The part of the Danish study used in this report comprised 1474 prevalent cases and 3466 controls.

**Norwegian study**

The Norwegian cases were recruited from the Oslo MS Registry^9^ and the controls were recruited from the Norwegian Bone Marrow Donor Registry. The cases were diagnosed in accordance with the Poser and/or McDonald criteria^10-11^ and informed written consent was obtained from all participants. The controls were matched to the cases by gender and age in five-year age strata at inclusion in the study.

Exposure data in the Norweigan study was collected in 2011 by sending an extensive questionnaire to potential participants. The participation rate was 70% for the cases and 84% for the controls. The time of the initial appearance of MS symptoms was used as an estimate of the disease onset, and the year in which this occurred was defined as the index year. The corresponding controls were given the same index year. Participants of non-Nordic origin were excluded as well as those who had stopped smoking before the index year. Cases who did not provide a blood sample were also excluded. Information regarding HLA genotype was available for 99% of the controls via the Norwegian Bone Marrow Donor Registry. In the Norwegian study, HLA-DRB1 genotypes were either obtained by a sequence based approach^12^ or imputed using HLA*IMP2^5^. The part of the Norwegian study used in this report comprised 211 prevalent cases and 692 controls.

**Serbian study**

Cases to the Serbian study were recruited at the Military Medical Academy, a military hospital open for civilians as well, and is the largest medical institution in the country. All patients fulfilled the McDonald criteria. Controls comprised of volunteers from employees of the Military Medical Academy (30%) and from the community (70%). Ethical approval was obtained by Ethical Committee of Military Medical Academy.

MS cases cared for at the Military Medical Academy completed a questionnaire by being personally interviewed by medical personnel. All invited patients accepted to participate in the study. Among potential controls, 92% agreed to participate. Controls answered the same questionnaire as the cases but filled it out at home. The recruitment of cases and controls took place during 2009 and 2010. All cases and controls who participated in the study provided a blood sample. Polymerase chain reaction (PCR) amplification with sequence-specific primers (Olerup SSP low resolution typing kits, Olerup SSP AB, Stockholm, Sweden) was used for genotyping participants in the Serbian study. In total, 457 cases and 505 controls from the Serbian study was included in the analysis.

**American study**The American case-control study is based on prevalent cases identified among members of Kaiser Permanente Medical Care Plan, Northern California Region (KPNC) using electronic medical records. All cases fulfilled the McDonald criteria. Controls were randomly selected from KPNC members and were individually matched to cases on gender, birth date, race/ethnicity, and zip code of the case residence. The study protocol was approved by the Institutional Review Boards of the KP Division of Research and the University of California, Berkeley.

Participants completed a computer-assisted telephone interview regarding life-style factors and various exposures. As of the data freeze in August 2014, the study included a total of 1479 cases and 1185 controls. Within this dataset, there were 1163 cases and 1178 matched controls. The study participation proportions were approximately 80% for cases and 66% for controls. Non-Caucasians and study participants who did not provide a blood sample were excluded. Participants from the American study were genotyped as previously described^13^. The part of the KPNC study used in this report comprised 1013 prevalent cases and 794 controls.

1. Diagnostic criteria for primary progressive multiple sclerosis: a position paper. Thompson AJ, Montalban X, Barkhof F, Brochet B, Filippi M, Miller DH, Polman CH, Stevenson VL, McDonald WI. Ann Neurol. 2000 Jun;47:831-835.
2. Hedström AK, Sundqvist E, Bäärnhielm M, et al. [Smoking and two human leukocyte antigen genes interact to increase the risk for multiple sclerosis.](http://www.ncbi.nlm.nih.gov/pubmed/21303861) Brain. 2011;134:653-64.
3. Hedström AK, Hillert J, Olsson T, Alfredsson L. Smoking and multiple sclerosis susceptibility. Eur J Epidemiol 2013;28:867-874.
4. Hedström AK, Bomfim IL, Barcellos LF, et al. Interaction between passive smoking and two HLA genes with regard to MS risk. Epidemiology 2014;43:1791-1798.
5. ?
6. EIMS genotypning. Dilthey 2013?
7. Hillert J, Stawiarz L. The Swedish MS registry - clinical support tool and scientific resource. Acta Neurol Scand Suppl 2015;132:11-19.
8. S, et al. Genetic risk and a primary role for cell-mediated immune mechanisms in multiple sclerosis. Nature 2011;476:214-219.
9. Smestad C, Sandvik L, Holmoy T, et al. Marked differences in prevalence of multiple sclerosis between ethnic groups in Oslo, Norway. J Neurol 2008;255:49-55.
10. Poser CM, Paty DW, Scheinberg L, et al. New diagnostic criteria for multiple sclerosis: guidelines for research protocols. Ann Neurol 1983;13:227-231.
11. Polman CH, Reingold SC, Banwell B, et al. Diagnostic criteria for multiple sclerosis: 2010 revisions to the McDonald criteria. Ann Neurol 2011;69:292-302.
12. Sayer et al. 2004?
13. Barcellos LF, Sawcer S, Ramsay PP, et al. Heterogeneity at the HLA-DRB1 locus and risk for multiple sclerosis. Hum Mol Genet 2006;15:2813-2824.
