# Supplementary material for: The interaction between smoking and HLA genes in multiple sclerosis: replication and refinement
Source: Eur J Epidemiol. 2017 Jun 8;32(10):909–19. doi: 10.1007/s10654-017-0250-2 (PMC5680370; doi:10.1007/s10654-017-0250-2)
Supplement: Supplementary file 2 — Supplementary material 2 (DOC 26 kb) [file 10654_2017_250_MOESM2_ESM.doc]

Supplementary table 2.

**Description of potential confouding variables adjusted for in EIMS and GEMS**

Residential area (county)

Passive smoking; dichotomized into ever or never exposed to passive smoking.

Snuff use; dichotomized into ever or never snuff users.

Alcohol consumption; categorized based on alcohol consumption at the index year (yes, no, or unknown).

Adolescent body mass index; calculated by dividing self-reported weight in kilograms by self-reported height in meters squared and adjusted for as a continuous variable.

Ultraviolet radiation exposure (UVR); based on three questions regarding exposure to UVR where each answer alternative was given a number ranging from 1 (the lowest exposure) to 4 (the highest exposure), we constructed an index by adding the numbers together and thus acquired a value between 3 and 12.

A history of infectious mononucleosis; dichotomized into yes or no.

Educational level; categorized into no post-secondary education, post-secondary education without university degree, or university degree.

Socioeconomic class; the last occupation during the year before the index year was used as a marker for socioeconomic class which was categorized into the following strata: 1, workers in goods production; 2, workers in service production; 3, employees at lower and intermediate levels; 4, employees at higher levels, executives, university graduates, and 5; others such as pensioners, students, and unemployed.
