# Supplementary material for: The interaction between smoking and HLA genes in multiple sclerosis: replication and refinement
Source: Eur J Epidemiol. 2017 Jun 8;32(10):909–19. doi: 10.1007/s10654-017-0250-2 (PMC5680370; doi:10.1007/s10654-017-0250-2)
Supplement: Supplementary file 3 — Supplementary material 3 (DOC 26 kb) [file 10654_2017_250_MOESM3_ESM.doc]

Supplementary table 3. OR with 95% CI of developing MS associated with smoking, absence of HLA-A*02 and HLA-DRB1*15, by study.

OR^1^ OR^2^ OR^3^ OR^4^ OR^5^ OR6^6^

Smoking 1.5 (1.3-1.8) 1.6 (1.4-1.8) 3.1 (2.7-3.5) 2.2 (1.5-3.1) 2.4 (1.8-3.1) 1.4 (1.1-1.8)

A2- 1.7 (1.5-2.0) 1.8 (1.6-2.0) 1.7 (1.5-2.0) 1.7 (1.2-2.5) 0.9 (0.7-1.2) 1.7 (1.4-2.2)

DR15 3.6 (3.1-4.1) 3.7 (3.3-4.1) 3.4 (3.0-3.9) 5.1 (3.6-7.4) 2.7 (2.0-3.7) 3.1 (2.4-3.9)

1=EIMS, Study 2=GEMS, 3=Danish study, 4=Norwegian study, 5=Serbian study, 6=American study

Each of the three risk factors were adjusted for the other two risk factors, as well as for age and gender.
