# Supplementary material for: The interaction between smoking and HLA genes in multiple sclerosis: replication and refinement
Source: Eur J Epidemiol. 2017 Jun 8;32(10):909–19. doi: 10.1007/s10654-017-0250-2 (PMC5680370; doi:10.1007/s10654-017-0250-2)
Supplement: Supplementary file 4 — Supplementary material 4 (DOC 54 kb) [file 10654_2017_250_MOESM4_ESM.doc]

**Supplementary table 4. EIMS April 2005-October 2008**

OR with 95% CI of developing MS for subjects categorized by HLA-DRB1*15 and HLA-A*02. Attributable proportion due to interaction between HLA-DRB1*15 and HLA-A*02.

**DR15+ A2- ca/co* OR (95% CI)# OR (95% CI)¤ p**
- - 76/321 1.0 (-) 1.0 (-)
- + 137/265 2.2 (1.6-3.1) 2.2 (1.6-3.1) <0.0001

+ - 142/134 4.5 (3.2-6.4) 4.5 (3.2-6.4) <0.0001
+ + 190/101 8.1 (5.7-11.6) 8.1 (5.7-11.5) <0.0001

AP 0.3 (0.07-0.5), p=0.01
* number of exposed cases and controls; # adjusted for age, gender, and smoking; **¤** adjusted for age, gender, smoking, passive smoking. snuff use, and alcohol habits.

OR with 95% CI of developing MS for subjects categorized by HLA-A*02 and smoking. Attributable proportion due to interaction between HLA-A*02 and smoking.
**A2- Smoking ca/co* OR (95% CI)# OR (95% CI)¤ p**
- - 126/291 1.0 (-) 1.0 (-)
- + 92/164 1.3 (0.9-1.8) 1.3 (0.9-1.9) 0.1
+ - 184/250 1.7 (1.3-2.3) 1.7 (1.3-2.3) 0.0003
+ + 143/116 3.2 (2.3-4.5) 3.3 (2.3-4.6) <0.0001
 AP 0.4 (0.1-0.6), p=0.002
* number of exposed cases and controls; # adjusted for age, gender, and HLA-DRB1*15; **¤** adjusted for age, gender, HLA-DRB1*15, passive smoking. snuff use, and alcohol habits.

OR with 95% CI of developing MS for subjects categorized by HLA-DRB1*15 and smoking. Attributable proportion due to interaction between HLA-DRB1*15 and smoking.

**DR15+ Smoking ca/co* OR (95% CI)# OR (95% CI)¤ p**
- - 114/387 1.0 (-) 1.0 (-)
- + 99/199 1.7 (1.2-2.4) 1.7 (1.3-2.4) 0.0008
+ - 196/154 4.4 (3.3-6.0) 4.4 (3.3-6.0) <0.0001
+ + 136/81 6.1 (4.3-8.6) 6.2 (4.3-8.8) <0.0001
 AP 0.2 (-0.1-0.5), p=0.3

* number of exposed cases and controls; # adjusted for age, gender, and HLA-A*02; **¤** adjusted for age, gender, HLA-A*02, passive smoking. snuff use, and alcohol habits.

**EIMS November 2008-December 2013**

OR with 95% CI of developing MS for subjects categorized by HLA-DRB1*15 and HLA-A*02. Attributable proportion due to interaction between HLA-DRB1*15 and HLA-A*02.

**DR15+ A2- ca/co* OR (95% CI)# OR (95% CI)¤ p**
- - 128/332 1.0 (-) 1.0 (-)
- + 194/385 1.3 (1.0-1.7) 1.3 (1.0-1.7) 0.05
+ - 193/192 2.6 (2.0-3.5) 2.6 (1.9-3.5) <0.0001
+ + 248/128 5.0 (3.7-6.8) 5.0 (3.7-6.8) <0.0001
 0.4 (0.2-0.6), p<0.0001
* number of exposed cases and controls; # adjusted for age, gender, and smoking; **¤** adjusted for age, gender, smoking, passive smoking. snuff use, and alcohol habits.

OR with 95% CI of developing MS for subjects categorized by HLA-A*02 and smoking. Attributable proportion due to interaction between HLA-A*02 and smoking.
**A2- Smoking ca/co* OR (95% CI)# OR (95% CI)¤ p**
- - 209/385 1.0 (-) 1.0 (-)
- + 112/139 1.5 (1.1-2.0) 1.4 (1.0-2.0) 0.03
+ - 286/380 1.5 (1.2-1.9) 1.5 (1.2-1.9) 0.0005
+ + 156/133 2.4 (1.8-3.2) 2.4 (1.8-3.2) <0.0001
 AP 0.2 (-0.1-0.5), p=0.2
* number of exposed cases and controls; # adjusted for age, gender, and HLA-DRB1*15; **¤** adjusted for age, gender, HLA-DRB1*15, passive smoking. snuff use, and alcohol habits.

OR with 95% CI of developing MS for subjects categorized by HLA-DRB1*15 and smoking. Attributable proportion due to interaction between HLA-DRB1*15 and smoking.
**DR15+ Smoking ca/co* OR (95% CI)# OR (95% CI)¤ p**
- - 212/524 1.0 (-) 1.0 (-)
- + 110/193 1.4 (1.1-1.9) 1.4 (1.0-1.8) 0.02
+ - 283/241 3.0 (2.4-3.8) 3.0 (2.4-3.8) <0.0001
+ + 158/79 5.2 (3.8-7.2) 5.0 (3.6-6.9) <0.0001
 AP 0.3 (0.1-0.5), p=0.004
* number of exposed cases and controls; # adjusted for age, gender, and HLA-A*02; **¤** adjusted for age, gender, HLA-A*02, passive smoking. snuff use, and alcohol habits.

**EIMS April 2005-December 2013**

OR with 95% CI of developing MS for subjects categorized by HLA-DRB1*15 and HLA-A*02. Attributable proportion due to interaction between HLA-DRB1*15 and HLA-A*02.

**DR15+ A2- ca/co* OR (95% CI)# OR (95% CI)¤ p**
- - 204/653 1.0 (-) 1.0 (-)
- + 331/650 1.6 (1.3-2.0) 1.6 (1.3-2.0) <0.0001
+ - 335/326 3.3 (2.7-4.1) 3.3 (2.7-4.1) <0.0001
+ + 438/229 6.2 (5.0-7.8) 6.2 (5.0-7.8) <0.0001

AP 0.4 (0.2-0.5), p<0.0001
* number of exposed cases and controls; # adjusted for age, gender, and smoking; **¤** adjusted for age, gender, smoking, passive smoking. snuff use, and alcohol habits.

OR with 95% CI of developing MS for subjects categorized by HLA-A*02 and smoking. Attributable proportion due to interaction between HLA-A*02 and smoking.
**A2- Smoking ca/co* OR (95% CI)# OR (95% CI)¤ p**
- - 335/676 1.0 (-) 1.0 (-)
- + 204/303 1.4 (1.1-1.7) 1.3 (1.1-1.7) 0.009
+ - 470/630 1.6 (1.3-1.9) 1.6 (1.4-1.9) <0.0001
+ + 299/249 2.8 (2.2-3.5) 2.7 (2.2-3.4) <0.0001 0.3 (0.1-0.5), p=0.002
* number of exposed cases and controls; # adjusted for age, gender, and HLA-DRB1*15; **¤** adjusted for age, gender, HLA-DRB1*15, passive smoking. snuff use, and alcohol habits.

OR with 95% CI of developing MS for subjects categorized by HLA-DRB1*15 and smoking. Attributable proportion due to interaction between HLA-DRB1*15 and smoking.
**DR15+ Smoking ca/co* OR (95% CI)# OR (95% CI)¤ p**
- - 326/911 1.0 (-) 1.0 (-)
- + 209/392 1.5 (1.2-1.9) 1.5 (1.2-1.9) <0.0001
+ - 479/395 3.5 (2.9-4.2) 3.5 (2.9-4.2) <0.0001
+ + 294/160 5.4 (4.3-6.9) 5.4 (4.3-6.9) <0.0001

0.3 (0.08-0.4), p=0.005
* number of exposed cases and controls; # adjusted for age, gender, and HLA-A*02; **¤** adjusted for age, gender, HLA-A*02, passive smoking. snuff use, and alcohol habits.

**GEMS**

OR with 95% CI of developing MS for subjects categorized by HLA-DRB1*15 and HLA-A*02. Attributable proportion due to interaction between HLA-DRB1*15 and HLA-A*02.

**DR15+ A2- ca/co* OR (95% CI)# OR (95% CI)¤ p**
- - 517/971 1.0 (-) 1.0 (-) 1.0 (-)
- + 700/649 2.0 (1.7-2.4) 2.1 (1.8-2.4) <0.0001
+ - 977/437 4.2 (3.6-5.0) 4.3 (3.7-5.0) <0.0001
+ + 1078/325 6.3 (5.4-7.4) 6.4 (5.5-7.6) <0.0001

AP 0.2 (0.04-0.3), p=0.01
* number of exposed cases and controls; # adjusted for age, gender, and smoking; **¤** adjusted for age, gender, smoking, passive smoking. snuff use, and alcohol habits.

OR with 95% CI of developing MS for subjects categorized by HLA-A*02 and smoking. Attributable proportion due to interaction between HLA-A*02 and smoking.
**A2- Smoking ca/co* OR (95% CI)# OR (95% CI)¤ p**
- - 777/890 1.0 (-) 1.0 (-)
- + 717/518 1.6 (1.4-1.8) 1.6 (1.4-1.9) <0.0001
+ - 971/637 1.8 (1.5-2.0) 1.8 (1.6-2.1) <0.0001
+ + 807/337 2.7 (2.3-3.2) 2.9 (2.4-3.4) <0.0001 AP 0.2 (0.008-0.3), p=0.04
* number of exposed cases and controls; # adjusted for age, gender, and HLA-DRB1*15; **¤** adjusted for age, gender, HLA-DRB1*15, passive smoking. snuff use, and alcohol habits.

OR with 95% CI of developing MS for subjects categorized by HLA-DRB1*15 and smoking. Attributable proportion due to interaction between HLA-DRB1*15 and smoking.
**DR15+ Smoking ca ca/co* OR (95% CI)# OR (95% CI)¤ p**
- - 622/1040 1.0 (-) 1.0 (-)
- + 595/580 1.7 (1.5-2.0) 1.7 (1.5-2.0) <0.0001
+ - 1126/487 3.9 (3.4-4.5) 4.0 (3.4-4.6) <0.0001
+ + 929/275 5.7 (4.8-6.7) 5.8 (4.9-6.9) <0.0001 AP 0.2 (0.06-0.3), p=0.005
* number of exposed cases and controls; # adjusted for age, gender, and HLA-A*02; **¤** adjusted for age, gender, HLA-A*02, passive smoking. snuff use, and alcohol habits.

**Danish study**

OR with 95% CI of developing MS for subjects categorized by HLA-DRB1*15 and HLA-A*02. Attributable proportion due to interaction between HLA-DRB1*15 and HLA-A*02.

**DR15+ A2- ca/co* OR (95% CI)# p AP**

- - 236/1297 1.0 (-)
- + 356/1118 1.8 (1.5-2.2) <0.0001
+ - 353/547 3.6 (3.0-4.4) <0.0001
+ + 529/504 5.9 (4.9-7.2) <0.0001 0.3 (0.1-0.4), p=0.0004
* number of exposed cases and controls; # adjusted for age, gender, and smoking;

OR with 95% CI of developing MS for subjects categorized by HLA-A*02 and smoking. Attributable proportion due to interaction between HLA-A*02 and smoking.

**A2- Smoking ca/co* OR (95% CI)# p AP**

- - 258/1323 1.0 (-)

- + 331/521 3.2 (3.6-4.0) <0.0001
+ - 414/1185 1.8 (1.5-2.1) <0.0001
+ + 471/437 5.4 (4.4-6.6) <0.0001 0.2 (0.005-0.3), p=0.04
* number of exposed cases and controls; # adjusted for age, gender, and HLA-DRB1*15;

OR with 95% CI of developing MS for subjects categorized by HLA-DRB1*15 and smoking. Attributable proportion due to interaction between HLA-DRB1*15 and smoking.
**DR15+ Smoking ca/co* OR (95% CI)# p AP**

- - 258/1748 1.0 (-)
- + 334/667 3.3 (2.7-3.9) <0.0001
+ - 414/760 3.6 (3.0-4.3) <0.0001
+ + 468/291 10.4 (8.5-12.8) <0.0001 0.4 (0.3-0.5), p<0.0001

* number of exposed cases and controls; # adjusted for age, gender, and HLA-A*02;

**Norwegian study**

OR with 95% CI of developing MS for subjects categorized by HLA-DRB1*15 and HLA-A*02. Attributable proportion due to interaction between HLA-DRB1*15 and HLA-A*02.

**DR15+ A2- ca/co* OR (95% CI)# p AP**

- - 34/270 1.0 (-)

- + 41/221 1.5 (0.9-2.5) 0.2
+ - 67/128 4.3 (2.6-7.2) <0.0001
+ + 69/73 8.9 (5.3-15.2) <0.0001 0.5 (0.2-0.7), p=0.0006
* number of exposed cases and controls; # adjusted for age, gender, and smoking;

OR with 95% CI of developing MS for subjects categorized by HLA-A*02 and smoking. Attributable proportion due to interaction between HLA-A*02 and smoking.
**A2- Smoking ca/co* OR (95% CI)# p AP**

- - 42/247 1.0 (-) 1.0 (-)
- + 59/151 2.0 (1.2-3.3) 0.003
+ - 46/195 1.6 (0.9-2.6) 0.07
+ + 64/99 3.9 (2.3-6.5) <0.0001 0.3 (0.0-0.7), p=0.04
* number of exposed cases and controls; # adjusted for age, gender, and HLA-DRB1*15;

OR with 95% CI of developing MS for subjects categorized by HLA-DRB1*15 and smoking. Attributable proportion due to interaction between HLA-DRB1*15 and smoking.
**DR15+ Smoking ca/co* OR (95% CI)# p AP**

- - 33/323 1.0 (-)
- + 42/168 2.6 (1.6-4.5) 0.0001
+ - 55/119 6.0 (3.5-10.3) <0.0001
+ + 81/82 11.7 (7.0-19.7) <0.0001 0.3 (0.05-0.6), p=0.02

* number of exposed cases and controls; # adjusted for age, gender, and HLA-A*02

**Serbian study**

OR with 95% CI of developing MS for subjects categorized by HLA-DRB1*15 and HLA-A*02. Attributable proportion due to interaction between HLA-DRB1*15 and smoking.
**DR15+ A2- ca/co* OR (95% CI)# p AP**

- - 148/211 1.0 (-)

- + 76/34 3.2 (2.0-5.0) <0.0001

+ - 141/206 1.0 (0.7-1.3) 0.9

+ + 92/54 2.4 (1.6-3.6) <0.0001 -0.3 (-1-0.4), p=0.4

* number of exposed cases and controls; # adjusted for age, gender, and smoking;

OR with 95% CI of developing MS for subjects categorized by HLA-A*02 and smoking. Attributable proportion due to interaction between HLA-A*02 and smoking.

**A2- Smoking ca/co* OR (95% CI)# p AP**

- - 62/105 1.0 (-)

- + 162/140 1.9 (1.3-2.8) 0.0009

+ - 55/123 0.8 (0.5-1.2) 0.2

+ + 178/137 2.2 (1.5-3.2) <0.0001 0.2 (-0.09-0.6), p=0.2* number of exposed cases and controls; # adjusted for age, gender, and HLA-DRB1*15;

OR with 95% CI of developing MS for subjects categorized by HLA-DRB1*15 and smoking. Attributable proportion due to interaction between HLA-DRB1*15 and smoking.

**DR15+ Smoking ca/co* OR (95% CI)# p AP**

- - 77/187 1.0 (-)
- + 212/230 2.2 (1.6-3.1) <0.0001
+ - 40/41 2.4 (1.4-4.0) 0.0009
+ + 128/47 6.5 (4.3-10.0) <0.0001 0.4 (0.2-0.7), p=0.0005

* number of exposed cases and controls; # adjusted for age, gender, and HLA-A*02

**KPNC study**

OR with 95% CI of developing MS for subjects categorized by HLA-DRB1*15 and HLA-A*02. Attributable proportion due to interaction between HLA-DRB1*15 and HLA-A*02.

**DR15+ A2- ca/co* OR (95% CI)# p AP**

- - 169/273 1.0 (-)

- + 302/302 1.6 (1.2-2.2) 0.001
+ - 198/119 2.5 (1.8-3.6) <0.0001
+ + 344/100 5.9 (4.1-9.3) <0.0001 0.5 (0.3-0.7), p<0.0001
* number of exposed cases and controls; # adjusted for age, gender, and smoking
;
OR with 95% CI of developing MS for subjects categorized by HLA-A*02 and smoking. Attributable proportion due to interaction between HLA-A*02 and smoking.
**A2- Smoking ca/co* OR (95% CI)# p AP**

- - 169/231 1.0 (-)

- + 198/161 1.9 (1.4-2.7) 0.0004
+ - 344/244 2.2 (1.6-3.0) <0.0001
+ + 302/158 3.1 (2.2-4.3) <0.0001 0.00 (-0.3-0.3), p=0.97
* number of exposed cases and controls; # adjusted for age, gender, and HLA-DRB1*15;

OR with 95% CI of developing MS for subjects categorized by HLA-DRB1*15 and smoking. Attributable proportion due to interaction between HLA-DRB1*15 and smoking.
**DR15+ Smoking ca/co* OR (95% CI)# p AP**

- - 233/342 1.0 (-)

- + 238/233 1.5 (1.1-2.0) 0.01
+ - 280/133 2.8 (2.1-3.8) <0.001
+ + 262/86 5.3 (3.7-7.6) <0.0001 0.4 (0.1-0.6), p=0.002
* number of exposed cases and controls; # adjusted for age, gender, and HLA-A*02;
